# Editorial: The origin and establishment process of gut microbiota in early life

**DOI:** 10.3389/fmicb.2023.1155660

**Published:** 2023-03-02

**Authors:** Yu Liu, Huixia Yang

**Affiliations:** ^1^Department of Obstetrics and Gynecology, Peking University First Hospital, Beijing, China; ^2^Beijing Key Laboratory of Maternal Fetal Medicine of Gestational Diabetes Mellitus, Beijing, China

**Keywords:** infant microbiota, early life, delivery mode, long-term health, metabolites

Early life represents a critical window of human growth and development, accompanied by the initial colonization and maturation of microbes (microbiota) and their genes (microbiome), which plays a crucial role in physiology, metabolism, nutrition, and immunity response. Maternal microbiome exerts key influence on early microbial establishment and maturation in infants. Through exposure to the birth cannel, postpartum breastfeeding and intimate skin contact, infant early microbiome is shaped by maternal microbiota from vaginal, fecal, skin, and breastmilk. In this specialized Research Topics collection of Frontiers in Microbiology, we investigate the establishment process and developmental trajectory of gut microbiota in early life, and the impact of maternal microbiota on the healthy conditions of offspring, such as metabolic diseases, food allergy, and growth development in both human and animal studies.

Several contributions in this Research Topics highlight the maternal microbiota and microbial metabolites on infant health. The review published Jiang et al. presented studies showing how researchers came to the path of investigating maternal-associated microbial metabolites and then to present studies linking them to the health conditions of offspring. Generally, most researchers investigated the possible relationship between gut microbiota alteration and risks of a wide range of diseases in the host, and following studies further found the associations may be mediated by the microbial metabolites, especially during pregnancy and lactation maternal-associated microbial metabolites may have crucial roles in participating the microbial regulation of offspring diseases development. However, few studies investigate the functions of maternal-associated metabolites. For the early prediction, early diagnosis, early prevention, or early treatment of child diseases, high-quality animal and clinical trials are needed. Also, studies comprehensively evaluating the effects of altered maternal-associated metabolites on overall maternal and infant health maintenance are required.

The study conducted by Wang S. et al. identify characteristics of the maternal gut microbiota in the third trimester and the infant gut microbiota in early life and the association of these microbiotas with infant food allergy. The results showed that maternal carriage of *Holdemania* during the third trimester strongly predicted the absence of food allergies in infants; However, this effect was not retained post-reconstruction of the infant gut microbiota after birth, suggesting that the effect of maternal gut microbiota on food allergy in the offspring may not be primarily mediated through the regulation of changes in the infant gut microbiota.

Preterm birth has an adverse effects of infant gut microbiota. Toubon et al. investigated the relationship between gut microbiota at 1 month after birth (hospitalization period) and 3.5 years of age in 159 preterm children. The results showed that the gut microbiota of preterm and full-term children at 3.5 years of age is characterized by two enterotypes dominated by either *Bacteroides* or *Prevotella*. Interestingly, we found that prematurity still imprints the gut microbiota of children, as the microbiota of preterm children showed lower diversity and different community composition than that seen in full-term children's microbiota. Additionally, the gut microbiota at 3.5 years of age was not related to that at 1 month in preterm children. At the same time, Chen et al. evaluate the characteristics of the gut microbiota of term Small for gestational age (SGA) infants and the associations between the gut microbiota in SGA infants and neurodevelopmental outcomes at 6 months of age. They found the gut microbial diversity of term SGA infants was significantly lower in the first week of life than that of term AGA infants. On the other hand, investigated microbial community assembly and dynamics in extremely low birth weight infants (ELBWI) over the first 2 weeks of life.

Maturation of the gut microbiota is shaped by numerous perinatal factors, and the delivery mode is a major determinant of the gut microbiota in the first weeks of life; cesarean section delivery disrupted the natural transmission of the gut microbiota from mothers to offspring. Matharu et al. highlight the strong impact of delivery mode on the gut microbiota developmental trajectories in healthy infants from 3 weeks to 1 year of age. The study shows a depletion of genus *Bacteroides* in 40% of the vaginally delivered infants result in the cluster to the cesarean section delivered infants, expanding the understanding of the impact of various early life factors on the colonization and dynamics of *Bacteroides* spp. in infants. In addition, the review submitted by Zhang et al. summarized the great significance of delivery mode on microbiota and health, as well as provided clinically feasible methods for the prevention and treatment of cesarean section related gut diseases.

This collection of articles provides an enhanced understanding of the developmental trajectory and effect factors of microbiota establishment in the early life, which also raises more questions. Studies comprehensively investigating the triangle relationship among the establishment of infant microbiota, various perinatal factors and long-term health maintenance are required.

## Author contributions

YL drafted the editorial text. All authors contributed to the article and approved the submitted version.

